# Free large sized intra-abdominal endometrioma in a postmenopausal woman: a case report

**DOI:** 10.1186/s12905-020-01054-x

**Published:** 2020-09-03

**Authors:** Antoine Naem, Anwar Shamandi, Ali Al-Shiekh, Bayan Alsaid

**Affiliations:** 1 Faculty of medicine of Damascus university, Damascus, Syria; 2 Al-Mouassat University Hospital, Damascus, Syria; 3 Al-Assad University hospital, Damascus, Syria

**Keywords:** Endometriosis, Endometrioma, Abdominal cyst, Omentum, Case report

## Abstract

**Background:**

Endometriosis is an estrogen-dependent disease defined by the presence of endometrial glands and stroma out of the uterine cavity. Its prevalence is estimated to be 2–10% in reproductive aged women. Endometriosis occurrence is estimated to be 2.55% in postmenopausal patients due to the decreased levels of estrogen. Endometriosis can present in three different forms: superficial peritoneal implants, ovarian endometriomas, and deep infiltrating endometriosis. Ovarian endometriomas are the most common form of endometriosis. Even though endometriomas have been encountered in various localizations, a free abdominal endometrioma was only reported once in a premenopausal patient. Here, we are reporting the first case of a free large endometrioma in a postmenopausal patient.

**Case presentation:**

A 67-year-old woman presented to the emergency department at our university hospital complaining of constipation and right flank pain. She suffered from uncontrolled primary hypertension and type 2 diabetes mellitus. On presentation, she was afebrile, hypertensive, and tachycardic. An abdominal CT scan revealed a large cystic mass measuring 17 × 26 cm in the anterior-posterior and transverse diameters respectively. The cyst caused bowel obstruction and right sided hydronephrosis. The patient underwent laparotomy and during the surgical exploration a large abdominal cyst adhered to the greater omentum was found. The cyst received plenteous blood supply from the greater omentum. The uterus and both ovaries were completely normal and didn’t have any connection to the cyst. An en-bloc cystectomy was performed successfully. The final histopathology report confirmed an abdominal endometrioma. The patient had an uneventful postoperative recovery.

**Conclusions:**

Endometriomas might reach large sizes regardless of their location or the patient’s age. The close relation of free abdominal endometriomas with the greater omentum suggests that these were developed from endometriotic omental implants. Endometrioma is rare in postmenopausal women. However, it should be considered as a possible differential diagnosis at any age.

## Background

Endometriosis is a chronic inflammatory disease defined by the presence of endometrial glands and stroma out of the uterine cavity. Its prevalence is estimated to be 2–10% in reproductive aged women [1], and up to 50% in infertile women [2]. However, its prevalence decreases sharply in postmenopausal women to 2.55% [3]. Endometriosis can take one of either three forms: superficial peritoneal implants, ovarian endometriomas, and deep infiltrating endometriosis [4]. Ovarian endometriomas were found to be the most common form of endometriosis [5]. Extra-pelvic localizations of endometriomas, such as umbilical endometriomas (Villar’s node) are not rare and could be also encountered [6]. However, a free abdominal endometrioma was only reported once in a reproductive aged patient [7]. Endometriosis pathogenesis is controversial. Many hypotheses have been proposed but none of them explains precisely the origin of each of the three endometriosis forms. The retrograde menstruation is the most accepted hypothesis. It states that endometrial cells travel backwards with the menstrual blood along the fallopian tubes to seed within the peritoneal cavity [8]. Even though endometriosis is asymptomatic in the majority of cases [9], it may negatively affect the quality of life of patients and interfere with their daily life activities when symptoms occur [10]. Endometriosis can also provoke dyspareunia that leads to sexual dysfunction in 2–4% of sexually active women [11]. Moreover, Endometriosis was reported to be the most common cause of chronic pelvic pain in female patients [12]. Endometriosis is an estrogen-dependent disease [12]. Therefore, menopause often results in complete remission of the endometriotic lesions and associated symptoms [13]. In some cases, endometriosis can be reactivated by the increased estrogen production from peripheral organs or under the influence of the hormone replacement therapy (HRT) [14]. Rarely, endometriosis can relapse in postmenopausal women with normal estradiol serum levels [15]. This is mainly attributed to the endometriosis ability of biosynthesizing estrogen via the aromatase activity [12, 16]. Here, we are reporting the case of a postmenopausal patient that presented with free abdominal endometrioma measuring 45 cm in its largest diameter. The large endometrioma manifested clinically as a cyst causing abdominal distention and bowel obstruction. Interestingly, the patient didn’t have a history of endometriosis. To the best of our knowledge, this is the first case of a free endometrioma that reached such a large size in a postmenopausal woman.

## Case presentation

**Fig. 1:**
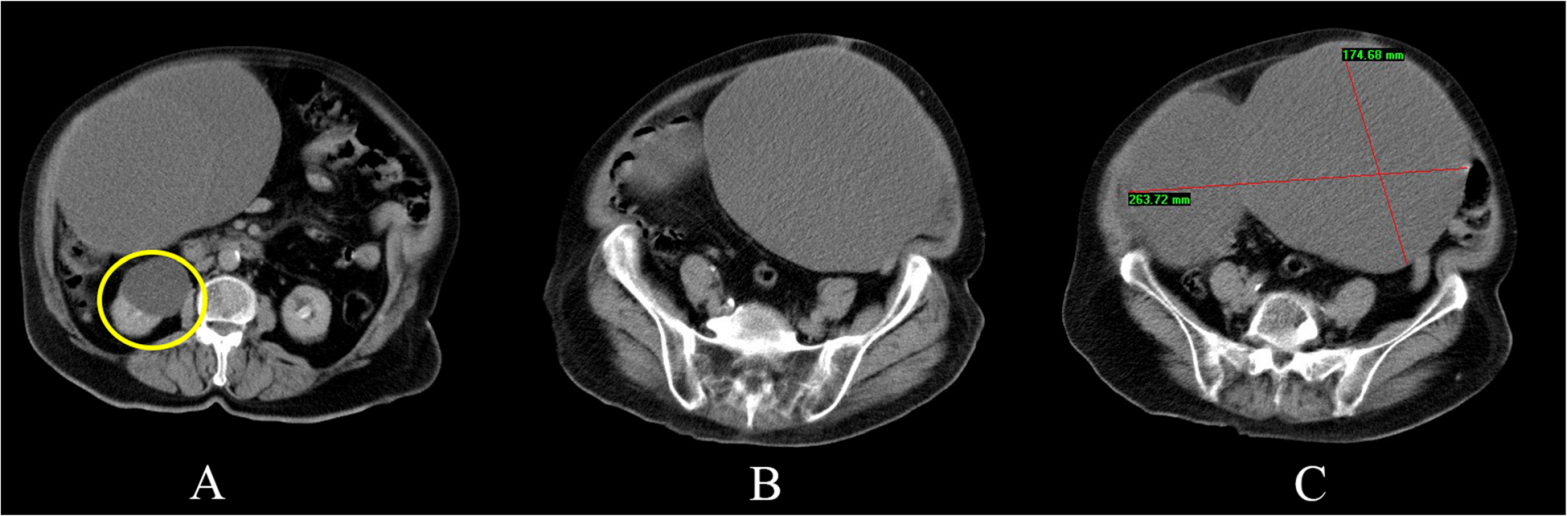
Computed tomography scan of the abdomen showing the homogenous cystic mass filled with isodense fluid. Note the presence of a right-sided hydronephrosis (yellow circle)

A 67-year-old woman presented to the emergency department at our university hospital complaining of constipation and right flank pain. She suffered from uncontrolled hypertension and type 2 diabetes mellitus. Additionally, the patient claimed that she was diagnosed with an adnexal cyst 7 years ago. Her previous gynecologist did not manage to determine exactly whether the cyst was ovarian or abdominal in origin and no further surgical interventions or sonographic imaging follow-up were carried out. On presentation, the patient was hypertensive and tachycardic. She was afebrile and her oxygen saturation was normal. Upon clinical examination, her abdomen was distended and non-tender. Her cardiac auscultation revealed arrhythmic arrhythmia. The laboratory tests were unremarkable except for a raised blood glucose level of 201 mg/dL. Her complete blood count, electrolytes, liver and kidney function tests were within the normal limits. A 12-leads electrocardiogram revealed atrial fibrillation with rapid ventricular response. A computed tomography scan (CT scan) of the abdomen revealed the presence of a large multilocular cystic mass measuring 17 X 26 cm in its anterior-posterior and transverse diameters respectively. The cyst occupied most of the abdomen and caused bowel obstruction and right sided hydronephrosis (Fig. 1).

**Fig. 2:**
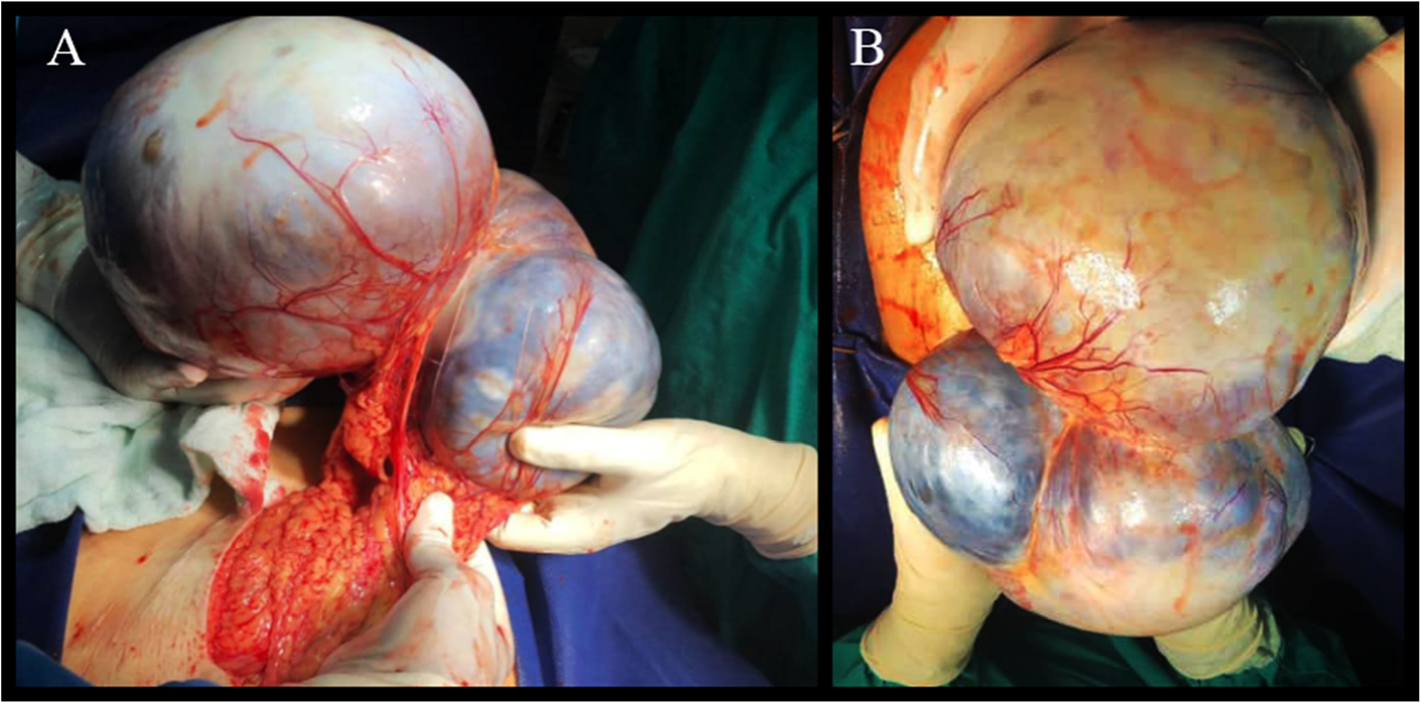
The gross appearance of the abdominal endometrioma. Note the extensive omental blood supply to the cyst

The initial differential diagnosis was either an omental cyst or abdominal abscess. The patient went preoperative optimization regarding the hypertension, hyperglycemia, and atrial fibrillation. The patient underwent a midline laparotomy. A large abdominal cyst adhered to the greater omentum was found. The cyst received its extensive vascular blood supply form the greater omentum (Fig. 2a). The uterus and both ovaries were unremarkable and were separate from the cyst. After ligating the cystic feeding vessels, an en-bloc cystectomy was performed successfully (Fig. 2b). Gross examination of the resected specimen revealed a large cystic mass measuring 45 cm in its longitudinal diameter and weighting 4.5 Kilograms. The cyst was multilocular and was filled primarily with clear fluid. Additionally, some cystic cavities were hemorrhagic.

**Fig. 3:**
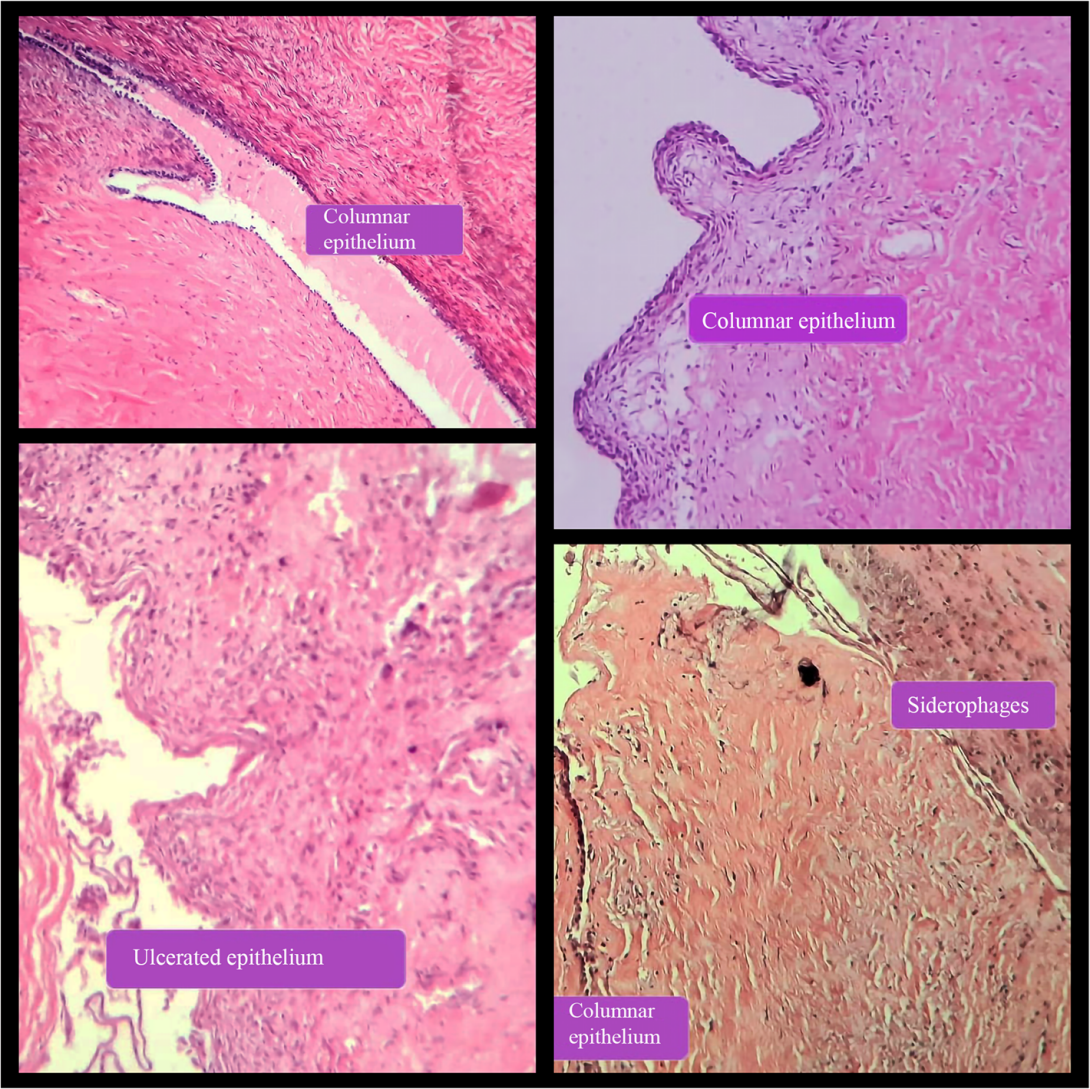
The microscopic appearance of the resected endometrioma stained in Hematoxylin & Eosin. Note the endometrial lining, endometrial stroma and siderophages

Microscopic examination of the cyst revealed a columnar endometrial lining with underlying endometrial stroma and siderophages (Fig. 3). Based on the cyst’s characteristics, an abdominal endometrioma was diagnosed. The patient was hospitalized for 4 days and her postoperative recovery period was uneventful.

## Discussion and conclusion

Endometriosis is known to be a disease of the reproductive age. This assumption may seem logic for the first instant regarding the substantial impact of estrogen on lesions’ progression. However, increasing data from the literature has shown that its occurrence in postmenopausal women is quiet possible. The most commonly seen form of endometriosis is ovarian endometriomas, also known as “chocolate cysts” [5]. Endometriomas form 27.2% of ovarian masses in women older than 40 years old, and only 4.3% of the ovarian pathologies in the sixth decade of life [17]. The relatively small number of endometriosis cases in postmenopausal women is primarily due to the diminished concentration of estrogen. After menopause, ovarian estrogen production declines significantly, which results in endometriosis remission in most patients [13]. However, endometriosis reactivation may result from the increased estrogen production from peripheral tissues –such as skin and fatty tissue- or by receiving HRT [14]. Nevertheless, endometriosis may present in the postmenopausal age without having a previous history of endometriosis. Regarding the remarkable percentage of asymptomatic cases, it’s unclear whether these lesions are undiagnosed premenopausal endometriosis or endometriosis originating in a postmenopausal age. The patient in this report did not receive HRT and unfortunately we did not measure her serum estradiol concentration to exclude the effect of endogenous estrogen. However, the presence of endometriosis in postmenopausal age without having previous HRT was reported in the literature [16]. This is mainly attributed to the endocrine intrinsic characteristics of endometriosis. Intrinsic signaling can make endometriotic cells function as independent unit. Ectopic endometrial cells can biosynthesize estrogen from cholesterol in the absence of any external substance, such as the adrenal androgens [12]. Endometriosis cells express a positive feedback loop that enhances the production of both estradiol E2 and prostaglandin E2 (PGE2) [12]. It is well established that PGE2 secreted from macrophages and ectopic endometrial cells stimulates the aromatase activity [18]. Estrogen in turn stimulates COX-2 enzyme to produce PGE2. It is estimated that retrograded endometrial cells duplicate the aromatase expression by 400 times [18, 19]. Apparently, the local estrogen production is as important as the circulating estrogen in fueling endometriosis. This is mainly evident in postmenopausal endometriotic patients with normal serum estrogen concentration [15]. Beside inducing ectopic endometrial cells proliferation, estrogen plays an important role in macrophage recruitment [20]. The recruited macrophages promote endometriosis progression and vascularization by secreting many growth factors, such as Transforming growth factor-β, Tumor necrosis factor-α, and Vascular endothelial growth factor (VEGF). It’s noteworthy that big amount of M2 macrophages was found within the endometriomas’ fluid in advanced stages of endometriosis. This finding attributes partially the fibrogenic effects of endometriosis to the activated M2 macrophages [21]. These may also contribute to the formation of the endometrioma’s fibrous wall. Moreover, growing evidence suggests that macrophages and other leukocytes secrete pro-inflammatory and adhesion molecules that alters the peritoneal environment. The aforementioned inflammatory changes provides better conditions for the differentiation, proliferation, adhesion and survival of the ectopic endometrial cells [22, 23]. The endometriotic cells also express VEGF intensively to maintain its vascularization [24]. It was postulated that the more endometriosis is well vascularized, the more active it becomes [25]. In fact, the plenteous blood supply that was provided by the omentum in our case and a previously reported one strongly support this postulation [7]. The extensive blood supply of the cyst may have contributed significantly to its massive enlargement. The exact formation mechanism of these cysts remains unknown. Some studies suggested the transformation of ovarian follicles and hemorrhagic corpora lutea into endometriomas [26, 27]. Nisolle et al. [4] suggested that endometriomas originate from the metaplastic transformation of the invaginated superficial ovarian coelomic epithelium. However, ovarian follicles and corpora lutea formation are directly linked to ovulation. The metaplastic transformation of the coelomic epithelium is achieved when it is exposed to estrogen concentration 10 times higher than the normal circulating estrogen level of the reproductive age [28]. Therefore, these mechanisms do not explain the endometrioma formation in our case. On the other hand, Brosens et al. [29] suggested that endometriomas are the result of adhesion formation between ovarian endometriotic implants, the peritoneum of the ovarian fossa and the broad ligament. This mechanism applies partly to our case because we believe that fibrogenesis around the endometrial cells within the omental substance is a substantial step in the pathogenesis of free abdominal endometriomas. The relation with the greater omentum may suggest a previous omental implantation of the endometriotic lesion, followed by the invasion of the submesothelial fatty tissue. The omentum can form a rich environment for endometriosis growth and progression. The omental fatty tissue provides the endometriotic cells directly with estrogen. We strongly suggest that free abdominal endometriomas are a special form of omental endometriosis. The low frequency of omental endometriosis –which is only 2%- may explain the rarity of this entity [30].

The preoperative diagnosis of endometriomas in unusual locations is challenging, especially when endometriomas reach large sizes. It is often confused with other neoplastic lesions that are more common in this group of patients, especially serous cystadenomas. These tumors are found to be the most common adnexal tumors in patients aged older than 60 years [17]. Different imaging modalities are useful to guide the pre-operative diagnosis. Endometrioma appearance on CT scan is nonspecific, and mimics many cystic masses. However, the presence of a central or peripheral hyper-dense focus within the cyst is a specific radiologic sign for endometriomas. Unfortunately, the sensitivity of this sign is estimated to be only 15% [31].

In reproductive aged women, endometriomas are usually treated by laparoscopic cystectomy. This approach carries a better pain relief and fertility outcomes compared to vaporizing the cystic cavity [32]. The recurrence rate after the cystectomy is estimated to be 10% [33]. Similarly, surgical resection of postmenopausal endometriosis remains the first line treatment. Unlike premenopausal endometriosis, postmenopausal endometriosis carries a higher risk of malignant transformation [34]. However, surgical interventions in patients older than 60 years often carry risks regarding the increased occurrence of chronic diseases. Therefore, alternative medical treatments should be considered. The third generation of aromatase inhibitors is highly recommended to treat postmenopausal endometriosis [35]. Administering letrozole or anastrozole for patients often resulted in symptoms relief within 4 to 15 months. Additionally, radiological regression of the disease was observed [34]. However, in these cases, endometriomas were generally small with good patients’ general condition. Often endometriomas reach large sizes due to self-neglect and lack of clinical follow-up. Therefore, early diagnosis of endometriomas by providing continuous counseling and keeping a high clinical suspicion might help patients to avoid potential risks of the surgical interventions.

In conclusions, endometriosis can affect women at any age. Although its occurrence is rare in postmenopausal women, it is still quite possible. Endometriomas might reach large sizes regardless their location or patient’s age. Free abdominal endometriomas are a unique manifestation of endometriosis. Its relation with the greater omentum might reflect the presence of previous endometrial implants on the omentum. Endometriosis should be considered as a possible differential diagnosis at any age. Good patient counselling and high clinical suspicion are key factors to diagnose postmenopausal endometriomas before reaching large sizes and provoking potential complications.

## Data Availability

All the relevant patient data and clinical history is provided within this article.

## Abbreviations

HRT: Hormone replacement therapy
CT scan: Computed tomography scan
PGE2: Prostaglandin E2
COX-2: Cyclooxygenase-2
VEGF: Vascular endothelial growth factor
